# European survey on imaging referral guidelines

**DOI:** 10.1007/s13244-013-0300-6

**Published:** 2013-12-13

**Authors:** Denis Remedios, Monika Hierath, Nick Ashford, Peter Cavanagh, Philippe A. Grenier, Catherine M. Lloyd, Georgi Simeonov, Julien-Aymeric Simonnet, Valérie Vilgrain

**Affiliations:** 1 Northwick Park Hospital, Harrow, UK; 2 European Society of Radiology (ESR), Vienna, Austria; 3 Royal College of Radiologists (RCR), London, UK; 4 French Society of Radiology (SFR), Paris, France; 5 European Commission Directorate-General for Energy (EC DG Energy), Brussels, Belgium

## Abstract

The objective of this study was to devise and implement a Europe-wide study on referral guidelines for radiological imaging in the EU Member States in order to identify potential major issues, important differences between Member States and good practices. A web-based survey was used to assess the availability of imaging referral guidelines, development methodology and preferences for future initiatives for European community action to facilitate justification and appropriate use of radiological diagnostic procedures. A questionnaire was distributed to representatives of national radiological and nuclear medicine societies as well as to competent authorities for radiation protection in 30 European countries, including all 28 EU Member States. Responses were collated and analysed to produce a series of conclusions and recommendations.

• *Survey respondents in 21/30 countries were aware of legal requirements for Guidelines*

• *Survey respondents in 18/30 countries were aware of the availability of Guidelines in their country.*

• *The majority of responders support the development of European Guidelines. These may either be from a combination of multiple national Guidelines with consensus or Pan-European Guidelines developed centrally.*

• *Guidelines developed in two countries included all of the following important features: radiation dose information; specific advice for imaging children; specific advice for the pregnant woman/unborn child; an evidence-based process; a formal consensus for recommendations.*

• *Suggestions for additional measures needed to reinforce the use of Guidelines include: educational initiatives; integrating Guidelines into clinical decision support systems; clinical audit for monitoring of the availability, use and implementation of Guidelines.*

**Electronic supplementary material:**

The online version of this article (doi:10.1007/s13244-013-0300-6) contains supplementary material, which is available to authorized users.

## Introduction

The European Council Directive 97/43/Euratom requires Member States “to ensure that recommendations concerning referral criteria for medical exposures, including radiation doses, are available to the prescriber of medical exposures” (Article 6.2 of Council Directive 97/43/EURATOM (Medical Exposures Directive, MED) [1].

Many Member States have developed or adopted national imaging referral guidelines (Guidelines) for clinical imaging, principally to support the referring practitioner in selecting and justifying radiological procedures. The selection of the appropriate investigation promotes good medical practice and radiation safety of patients. Guidelines have been available in Europe since 1989 when the Royal College of Radiologists (RCR) first published “Making the best use of a department of clinical radiology” [2] in the UK. The Radiation Protection 118: Referral Guidelines for Imaging (RP 118) [3] was published in 2000 by the European Commission (EC). The French Society of Radiology (SFR) developed and published their imaging referral guidelines, “Guide du bon usage des examens d'imagerie médicale” in 2005, updated in 2012 [4].

The EC-sponsored project “Implementation of Council Directive 97/43/Euratom requirements concerning referral criteria for medical imaging in the European Union” was carried out by a consortium of European partners starting with this survey of the availability of Guidelines in 2012.

The consortium partners are:

- European Society of Radiology (ESR) www.myesr.org
- Royal College of Radiologists (RCR) www.rcr.ac.uk French Society of Radiology (SFR) www.sfrnet.org
- Cardiovascular and Interventional Radiology Society of Europe (CIRSE) www.cirse.org
- European Society of Paediatric Radiology (ESPR) www.espr.org

This paper outlines the development, implementation, results and conclusions drawn from the survey on imaging referral guidelines for radiological imaging in 30 European countries. The aim of this study was to identify the current position of European countries with respect to referral guidelines, to identify good practices in guideline development and implementation and to canvass opinion as to the way forward. A web-based survey was used to assess the availability of Guidelines, development methodology and preferences for future initiatives for European community action to facilitate justification, and to implement appropriate use of diagnostic and interventional radiological procedures.

## Methods

A web-based questionnaire was drafted by the RCR, together with other members of the Referral Guidelines Project Consortium, and distributed to representatives of national radiological and nuclear medicine societies, as well as to representatives of national competent authorities responsible for radiation protection, in 30 European countries including 28 EU Member States and two countries implementing EU legislation—Norway and Switzerland.

Attention was paid to ensure delivery to the nominated representative, to avoid duplication and to encourage timely response to the questionnaire. Address lists were obtained from the ESR, EC Directorate General for Energy and the European Association of Nuclear Medicine (EANM). The EC Directorate General for Energy kindly assisted in encouraging completion of the questionnaire by competent authority representatives.

The design of the questionnaire was based on three sections:

1. Information regarding guideline availability, legal requirements and imaging requesting issues.
2. Methodology for development and distribution of guidelines.
3. Preferences for future guideline development, format, distribution, tools for reinforcement and monitoring.

To avoid bias and to facilitate analysis, input from an experienced psychologist was used. The survey was compiled in SurveyMonkey™ (SurveyMonkey, Palo Alto, California, USA. www.surveymonkey.com). It was designed to be easily understood with carefully chosen, unambiguous terminology and logical flow which led responders through only the relevant questions. The design facilitated statistical analysis, limiting the number of free text answers.

Survey responses were collated by the ESR and analysed by the SFR. A balanced seven-point Likert scale was used to quantify the answers to those questions involving levels of agreement or support. A 75 % or greater level of agreement was considered to represent consensus.

## Results

### Participation

Responses were received from representatives of all 30 countries surveyed. Eighty responses were received:

- 32 from national radiological societies (some countries had more than one national society)
- 20 from national nuclear medicine societies
- 28 from national competent authorities

Regarding the radiological societies, multiple responses were received from The Netherlands (two) and Romania (three). No response was received from Cyprus.

Regarding the national nuclear medicine societies, three responses were received from The Netherlands. No response was received from Bulgaria, Estonia, Finland, France, Ireland, Lithuania, Luxembourg, Norway, Poland, Portugal, Slovakia or Slovenia.

Regarding the national competent authorities, two responses were received from Spain, but none from Italy, Hungary and Latvia.

In the analysis, all the responses received were taken into account using the majority response (rounding up) for those countries with multiple responses.

### Availability of imaging referral guidelines in European countries

Sixty-one percent of responders said that there was a legal requirement for Guidelines including radiation dose. Five responders were uncertain as to the existence of such legislation. It was confirmed at a later date that all EU member states had such a requirement.

Most respondents report that the responsibility for making Guidelines available has been transferred to ministries of health. There was some discordance between national societies and competent authorities’ responses to the question about the transfer of responsibility for making Guidelines available.

The majority of respondents (76 %) did not think that Guidelines must exist in order for insurance companies in their country to pay for an imaging investigation. Respondents indicated that most imaging requests are made by medical practitioners. Respondents report that general practitioners make more requests for plain radiographs, contrast radiography, and ultrasound (US), and fewer for computed tomography (CT), magnetic resonance imaging (MRI), interventional radiology and nuclear medicine examinations compared with hospital specialists.

Respondents indicated that the most common modality for which a patient could self-present was US, followed by plain radiography and MRI.

Twenty radiology societies, 12 nuclear medicine societies and eight competent authorities responded that there were nationally recognised imaging referral guidelines including radiation dose available.

Analysis by country showed that representatives in 70 % of European countries were aware of the legal requirement for guidelines and respondents in 60 % of countries were aware of the presence of referral guidelines [2, 4–24] (*see* Table 1).

**Table 1:** Guidelines survey: national radiology society, nuclear medicine society and competent authority responses to the availability of and legal requirements for imaging referral guidelines including radiation doses

	Does your Member State: have a legal requirement for imaging referral guidelines including radiation doses (“Guidelines”)				In your Member State, are there nationally recognised imaging referral guidelines (appropriateness or referral criteria) including radiation doses available?			
Country	Comp auth	NM Soc.	Rad Soc.	Majority response^c^	Comp auth	NM Soc.	Rad Soc.	Majority response
Austria	yes	yes	yes	**yes**	yes	yes	yes	**yes**
Belgium	yes	yes	no	**yes**	yes	yes	yes	**yes**
Bulgaria	yes	-	no	**no**	yes	-	yes	**yes**
Croatia^a^	no	no	yes	**no**	no	no	yes	**no**
Cyprus	yes	yes	-	**yes**	no	no	-	**no**
Czech Republic	yes	yes	yes	**yes**	yes	yes	no	**no**
Denmark	yes	don’t know	yes	**yes**	no	yes	yes	**yes**
Estonia	no	-	yes	**no**	no	-	yes	**no**
Finland	yes	-	yes	**yes**	yes	-	yes	**yes**
France	yes	-	yes	**yes**	yes	-	yes	**yes**
Germany	yes	yes	yes	**yes**	yes	yes	yes	**yes**
Greece	yes	yes	yes	**yes**	yes	no	yes	**yes**
Hungary	-	yes	no	**yes**	-	yes	no	**yes**
Ireland	yes	-	yes	**yes**	yes	-	-	**no**
Italy	-	yes	yes	**yes**	-	yes	yes	**yes**
Latvia	-	yes	yes	**yes**	-	yes	yes	**yes**
Lithuania	no	-	no	**no**	no	-	no	**no**
Luxembourg	yes	-	no	**yes**	yes	-	yes	**yes**
Malta	no	yes	yes	**yes**	no	no	no	**no**
Netherlands	no	yes	yes	**yes**	no	yes	yes	**yes**
Norway^b^	no	-	no	**no**	yes	-	no	**no**
Poland	yes	-	yes	**yes**	no	-	yes	**yes**
Portugal	no	-	yes	**yes**	no	-	no	**no**
Romania	yes	yes	yes	**yes**	no	yes	yes	**yes**
Slovakia	yes	-	yes	**yes**	yes	-	yes	**yes**
Slovenia	yes	-	no	**yes**	no	-	yes	**no**
Spain	No	no	no	**no**	no/-	yes	yes	**yes**
Sweden	yes	no	yes	**no**	yes	no	no	**no**
Switzerland^b^	yes/no	yes	no	**no**	no	yes	-	**no**
United Kingdom	yes	don’t know	yes	**yes**	yes	no	yes	**yes**
Overall yes	19	13	22	**21**/**30**	14	12	23	**18**/**30**
Overall no	9	4	10	**9**/**30**	13	6	7	**12**/**30**

Thirty European countries took part in the survey, including 27 EU member states, one acceding state^a^ and two countries enacting EU legislation^b^

^c^Subsequent to the survey, all EU countries have confirmed that they have a legal requirement for referral guidelines

### Guideline methodology

Only 12 of the 20 radiological societies and 9 of the 12 nuclear medicine societies which have nationally recognised imaging referral guidelines including radiation dose answered the subsequent questions concerning specifically the content of these Guidelines. The replies for this section on guideline methodology may not be fully representative of the 30 European countries as only 23 responses from 17 countries were received out of 52 professional society representatives (and a total of 80 respondents including competent authority representatives).

### Preference for source of Guidelines to be used in Europe

**Fig. 1:**
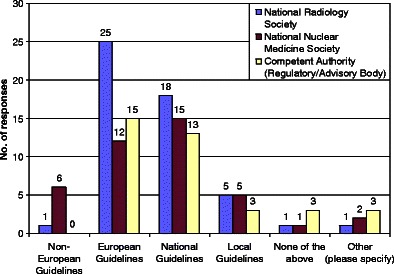
Availability of Guidelines. Preference for the source of referral guidelines to be used in Europe

The great majority of states have recommended European or national Guidelines (*see* Fig. 1).

Where “other” was chosen as the question response, the specified other options for the preference of the source of Guidelines included:

“The professional societies are responsible for drawing up the referral guidelines.”

“Our government would most probably accept competent international guidelines and the Croatian Society of Radiology is currently trying to introduce Royal College of Radiologists’ guidelines in our clinical practice modified according to our situation.”

“Current regulation does not [recommend any guidelines] but new draft regulation and guidelines recommend European Guidelines.”

“European guidelines are to be recommended in new legislation.”

“If National guidelines are not available then European ones are implemented.”

“American guidelines (US).”

### Development of Guidelines

**Fig. 2:**
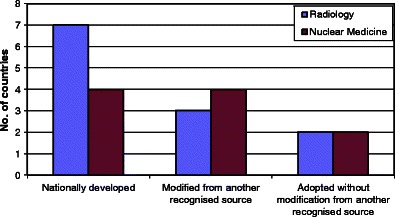
Guidelines are either developed de novo nationally, adopted and adapted from other sources or adopted without adaptation from other sources. Twenty-two responses from 14 countries were received out of a total of 52 professional organisations

Guidelines were reported (by respondents from just 14 countries) to be developed nationally in half of the countries and modified or adopted with modifications from another source in the others (*see* Fig. 2).

There was a good concordance between radiology and nuclear medicine societies for two countries (France and UK), which provided radiological and nuclear medicine guidance within the same publication.

The year of the first edition of the Guidelines varied from 1989 to 2005 for radiological societies and from 1998 to 2011 for nuclear medicine societies. The approximate duration of the review cycle has varied between countries from 3-4 years to >6 years.

In the majority of countries (67 % for radiology and 90 % for nuclear medicine), the source of funding for the development of the Guidelines was the Ministry of Health or other governmental department.

### Imaging modalities included in Guidelines

The imaging modalities involving ionising radiation (radiography and nuclear medicine) were included in the great majority of Guidelines (83-92 %), whereas the non-ionising radiology modalities (US, MRI) were present in only 75 % of Guidelines.

### Guidance for children and for pregnant women

The majority of National Guidelines include separate guidance for children (67 % for radiology and 80 % for nuclear medicine) and pregnant women (83 % for radiology and 78 % for nuclear medicine).

### Focus of Guidelines on clinical presentations or indications for procedures

Most radiological Guidelines focus on clinical presentations, whereas most nuclear medicine guidelines focus on indications for procedures. Most of the radiological societies’ Guidelines and the nuclear medicine societies’ Guidelines cover multiple groups of diseases and medical conditions for adults, including: breast, cancer, cardiovascular, chest, gastrointestinal, neurological, trauma and urogenital. Guidelines for children cover fewer clinical conditions.

The radiological societies’ Guidelines included between 200 to 500 clinical conditions or diagnostic problems, and the nuclear medicine societies’ Guidelines between 16 and 300.

### Use of evidence levels and recommendations

Very few Guidelines have included recognised evidence levels (six radiology, three nuclear medicine) and grading recommendation using a recognised system (four radiology, one nuclear medicine).

Radiation dose, strength of evidence and grading of recommendations were considered in most Guidelines. Cost effectiveness and availability of equipment or expertise were far less frequently taken into consideration.

Two radiological societies graded their recommendations (France and UK). Both used the grades of recommendation as defined by The Oxford Centre for Evidence-Based Medicine—Evidence levels and grades of recommendations, 2009 [25]. In the French Society of Radiology Guidelines there were 62 grade A recommendations, 618 grade B recommendations and 209 grade C recommendations. In the RCR Guidelines from the UK there were 74 grade A recommendations, 633 grade B recommendations and 166 grade C recommendations.

### Use of a recognised process of consensus

Delphi process was used in three radiological societies’ Guidelines (Finland, France, UK). Expert meeting for consensus was used by four radiological societies and five nuclear medicine societies.

### Use of recognised sources for radiation dose and costs

Radiation dose was obtained from recognised sources in eight radiological and nine nuclear medicine societies.

### Format of Guidelines

**Fig. 3:**
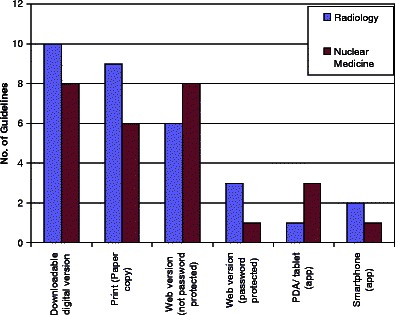
Most guidelines are available in downloadable digital, print and online versions

Regarding the format and dissemination of the National Guidelines (Fig. 3), almost all Guidelines are available in a downloadable digital version. The great majority of Guidelines are available in a web version. Very few have a tablet or smart phone version.

### Distribution of Guidelines

The majority of Guidelines are routinely circulated to providers of the service, general practitioners, emergency department clinicians and specialists/hospital doctors. Only a few of these Guidelines are routinely circulated to non-healthcare professionals, medical students, funders and the public.

### Reinforcement of Guidelines

Reinforcement of Guidelines is advocated through periodical reminders in half and through educational message by most of the radiological societies.

Guidelines are also used for education and academic research.

### Wishes for the use of Guidelines in Europe

For the remaining items in the survey, respondents were asked to rate agreement on a seven-point balanced Likert scale regarding preferences for European Guideline development, format, media, barriers and their solutions, and methods for monitoring. There were 28 responses from radiology societies, 18 from nuclear medicine societies and 27 from competent authorities. All responses were taken into account. Positive responses rated 5-7 on a seven-point scale were taken as agreement and consensus considered strong where there was agreement by at least 75 % of respondents.

### European preferences for Guidelines

**Fig. 4:**
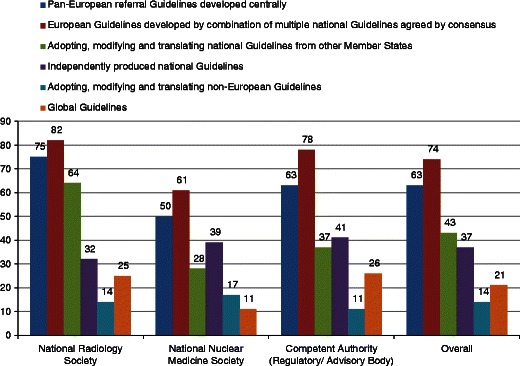
Preferences for the future of imaging referral guidelines in Europe. European Guidelines preferably developed by combination of National Guidelines were favoured with high-level agreement (Likert scores of 5-7 out of 7) among Radiology Societies and Competent Authorities

Eighty-two percent of radiology societies and 78 % of competent authorities support European Guidelines developed by a combination of multiple national Guidelines agreed by consensus. This is also supported to a lesser extent by nuclear medicine societies (61 %). Seventy-five percent of radiology societies support Pan-European Guidelines developed centrally (*see* Fig. 4).

### Guideline format

**Fig. 5:**
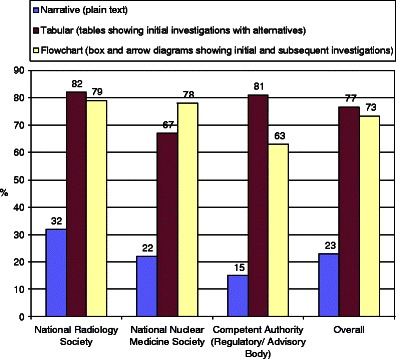
Guidelines survey: format. Tabular and flow chart formats are preferred, both reaching a high level of consensus at Likert 5-7/7 scores

Most societies and competent authorities support tabular and flowchart format for the Guidelines (*see* Fig. 5).

### Media and mode for distribution

**Fig. 6:**
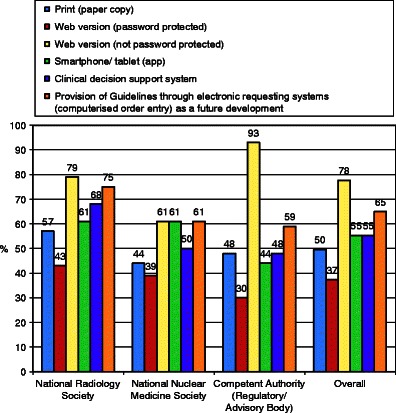
Guidelines survey: distribution. Although multiple media are favoured, a web version is the preferred basic medium. Provision of guidance through electronic requesting systems (such as an open architecture decision support system) has good support reaching strong consensus among radiological societies

Most societies and competent authorities support an open web version for distribution mode. Seventy-five percent of radiology societies support provision of Guidelines through electronic requesting systems as a future development (*see* Fig. 6).

### Potential barriers/challenges to the effective distribution of Guidelines

**Fig 7:**
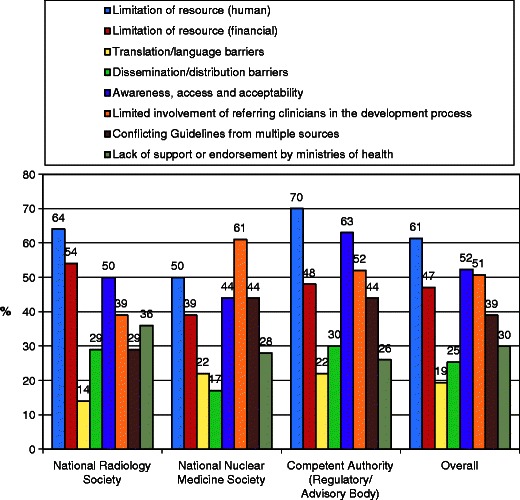
Guidelines survey: potential barriers to distribution. Agreement at Likert 5-7/7 for resource limitation, limited awareness and limited clinician involvement are common barriers. Translation and language issues are not perceived by many to be a barrier

Resource limitation, limited awareness and limited clinician involvement are common barriers to the effective distribution of Guidelines (*see* Fig. 7).

### Suggestions of solutions to barriers limiting the availability of Guideline use

**Fig. 8:**
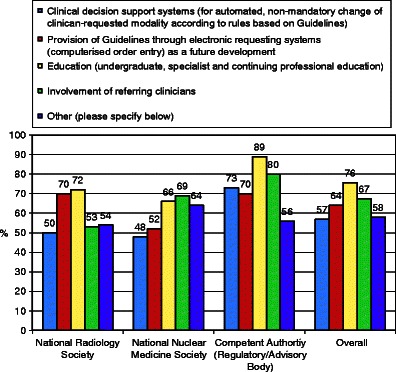
Guidelines survey: suggested solutions to barriers. Educational initiatives are slightly favoured overall

Education and involvement of referring clinicians are mostly proposed by competent authorities as potential solutions to overcome the barriers limiting the availability of Guidelines (*see* Fig. 8).

### Preferred methods for monitoring Guideline use

**Fig. 9:**
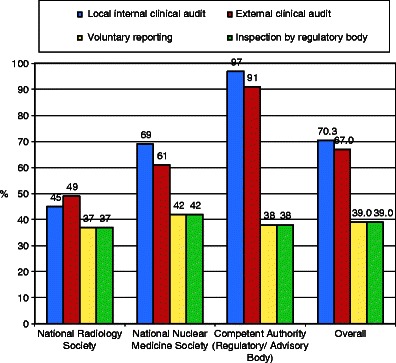
Preferred methods for monitoring use of imaging guidelines. Clinical audit, both internal and external, is favoured, particularly by competent authorities

All respondents and particularly competent authorities strongly support local internal and external clinical audits to monitor Guideline use (*see* Fig. 9).

## Discussion

The results of the initial survey indicated that imaging referral guidelines are available in two-thirds of the EU Member States with a legal requirement for guidelines, and only in one-third of countries who do not have a legal requirement for guidelines.

It is recognised that a limitation of this study is the paucity of responses to the section of the survey dealing with guideline methodology. This may be partly explained by the popular principle of adopting and adapting Guidelines from other countries. Furthermore discordance in replies from representatives of professional societies and competent authorities from within a country may be due to limited awareness of the current position in a specialised area. At the Project Workshop in Vienna, Austria, September 2012, participants considered that limited awareness, rather than limited availability, may have been a reason for the reported availability of legal requirements and Guidelines availability.

The decision was made to re-audit the legal requirement for and availability of referral guidelines in those countries previously reporting that referral guidelines were not available. In addition it was felt that the encouragement of good practices shared through the workshop and survey, together with facilitation of processes for adopting, adapting and translating referral guidelines, may have had a positive influence.

To update the situation, those countries previously reporting no guideline availability were re-audited with surveys sent out to national professional societies and competent authorities enquiring as to the awareness of availability of national legislation and to the availability of referral guidelines, including work in progress.

This re-audit showed that since the initial survey, carried out during the spring of 2012, a further seven countries had either begun measures to make referral guidelines available or had identified existing national referral guidelines, bringing the number of countries with such guidelines available to 25 out of a total of 30.

In addition, the re-audit confirmed awareness of a legal requirement for referral guidelines in all 30 countries targeted by the survey.

## Conclusions

The following conclusions were made from the Guidelines survey based on responses from radiological societies, nuclear medicine societies and radiological competent authorities:

### Availability of Guidelines

1. Sixty percent of respondents indicated that they have a legal requirement for imaging referral guidelines including radiation doses.
2. Survey respondents in 21/30 countries were aware of legal requirements for Guidelines.
3. Twenty-three national radiology societies (77 %), 12 national nuclear medicine societies (66 %) and 14 competent authorities (52 %) indicated that there are nationally recognised imaging referral guidelines including radiation doses available.
4. Survey respondents in 18/30 countries were aware of the availability of Guidelines in their country.
5. From responses, two out of three countries with a legal requirement for Guidelines have Guidelines available, whereas only one in three countries without a legal requirement has Guidelines available.

### Guidelines development methodology

1. Responses to survey questions concerning Guideline methodology may not be fully representative of the whole EU as only 23 respondents (out of 80 in total) from 17/30 countries replied to this section. The low response rate is in part due to the popular principle of adoption.
2. The majority of responders support the development of European Guidelines. These may either be from a combination of multiple national Guidelines with consensus or Pan-European Guidelines developed centrally.
3. Not all national Guidelines available are based on clinical presentations. Some nuclear medicine Guidelines are based on indications.
4. Good practices were demonstrated in several countries which included some of the important methodological features shown below. Guidelines developed in two countries included all of these features:
  - Radiation dose information
  - Specific advice for imaging children
  - Specific advice for the pregnant woman/unborn child
  - An evidence-based process
  - Formal consensus for recommendations.

### Suggestions for initiatives for improving the use of Guidelines

1. Agreement that additional measures were needed to reinforce the use of Guidelines.
2. Educational initiatives are highly favoured to improve implementation.
3. There is strong support for the concept of integrating Guidelines into clinical decision support systems.
4. Clinical audit should be used for monitoring of Guidelines’ availability, their use and implementation.

## Electronic supplementary material

Below is the link to the electronic supplementary material.

ESM 1 (PDF 442 kb)
