# Supplementary material for: European survey on imaging referral guidelines
Source: Insights Imaging. 2013 Dec 13;5(1):15–23. doi: 10.1007/s13244-013-0300-6 (PMC3948911; doi:10.1007/s13244-013-0300-6)
Supplement: Supplementary file 1 — (PDF 442 kb) [file 13244_2013_300_MOESM1_ESM.pdf]

European Society of Radiology

EC Survey on Imaging Referral Guidelines

March 27-April 27, 2012

PDF file for information only
Please fill in survey online at
https://www.surveymonkey.com/s/EC_ImagingReferralGL_Survey

In case of questions, please contact
Denis Remedios (denis.remedios@imperial.ac.uk)
Monika Hierath (monika.hierath@myesr.org)

EC Survey on Imaging Referral Guidelines

Availability of Guidelines

IMPORTANT INFORMATION TO SAVE YOU TIME. PLEASE READ FOLLOWING BEFORE STARTING SURVEY.

Imaging referral guidelines are useful in the justification and selection of appropriate radiological and nuclear medicine procedures, help with radiation safety of patients and may enhance cost-effectiveness in health organisations. The European Society of Radiology is leading a project (together with the RCR, SFR, CIRSE and ESPR) sponsored by the European Commission which will guide initiatives to improve the availability of imaging referral guidelines in Europe.

The Council Directive 97/43/Euratom stipulates that member states shall ensure that recommendations concerning referral criteria for medical exposure, including radiation doses, are available to the prescribers of medical exposure.

[Euratom ref. http://ec.europa.eu/energy/nuclear/radioprotection/doc/legislation/9743_en.pdf]

This survey is to assess the current situation in EU member states. Please fill in the survey on behalf of your Organisation. Responses will be treated confidentially.

Representatives of competent authorities (regulatory bodies) are requested to answer only starred questions and need not answer questions regarding guideline methodology.

Technical instructions:
- The survey does not have to be completed in one sitting; it can be saved and re-accessed via the link provided in the invitation email at any point of time until the closing date. Please complete the survey at the SAME WORKPLACE (same IP address) and within the SAME BROWSER in which it was started. This is necessary to identify you correctly as the same participant.
- Every given answer is saved immediately, therefore no replies will be lost if the survey is not completed in one sitting
- Answers can be amended until the closing date of the survey; please just re-enter the survey and select the new answer for the concerned question(s).
- Square check boxes stand for questions that allow multiple answers to be selected; round radio buttons only enable the selection of 1 answer per question.
- Starred questions (*) are mandatory.
- For navigation, please use the two buttons (PREV or SAVE AND NEXT) at the bottom of the page, NOT the arrows on the browser".

Please fill in all full-text answers in English.

The closing date of the survey is April 27, 2012.
If you have any questions or difficulties, please contact Ms. Monika Hierath, Project Manager (monika.hierath@myesr.org).

Thank you for your time and effort!

***1. Please enter your email address in case of queries or for clarification:**

Availability of Guidelines

***2. Please indicate if you are responding on behalf of a**

- National radiology society
- National nuclear medicine society
- Competent authority (regulatory/advisory body)

***3. Please give the name in English of the organisation on whose behalf you are completing the questionnaire:**

***4. Please indicate your Member State / Country:**

***5. Does your Member State have a legal requirement for imaging referral guidelines including radiation doses ("Guidelines")?**

- Yes
- No
- Don't know

***6. Does your Member State transfer responsibility for making Guidelines available?**

- Yes
- No
- Don't know

Availability of Guidelines

*7. To whom is responsibility transferred?

- [ ] Departments/ministries of health (competent authorities)
- [ ] Health organisations
- [ ] Hospital/employers
- [ ] Professional organisation
- [ ] Referring medical practitioners
- [ ] Other (please specify)

Availability of Guidelines

*8. Does your Member State recommend (please tick all that apply):

- [ ] Non-European Guidelines
- [ ] European Guidelines
- [ ] National Guidelines
- [ ] Local Guidelines
- [ ] None of the above
- [ ] Other (please specify)

Availability of Guidelines

9. Please specify which Non-European Guidelines your Member State recommends:

Availability of Guidelines

***10. Are there any national or specific insurance requirements stating that a Guideline must exist in order for there to be a payment for an imaging investigation (in either the public or private sectors)?**

- ◯ Yes (please specify below)
- ◯ No
- ◯ Don't know

If yes, please specify requirements (particularly regarding pre-authorisation of procedures by insurers):

***11. In your Member State, for which modalities can the following groups of healthcare professionals make requests:**

	Plain radiography	Radiographic contrast procedure	US	CT	MRI	Interventional radiology	PET-CT	Nuclear medicine
General practitioner	☐	☐	☐	☐	☐	☐	☐	☐
Emergency dept. clinician	☐	☐	☐	☐	☐	☐	☐	☐
Specialist / hospital doctor	☐	☐	☐	☐	☐	☐	☐	☐
Chiropractor	☐	☐	☐	☐	☐	☐	☐	☐
Nurse	☐	☐	☐	☐	☐	☐	☐	☐
Physiotherapist	☐	☐	☐	☐	☐	☐	☐	☐
Other [please specify below]	☐	☐	☐	☐	☐	☐	☐	☐

Other

***12. In your Member State, for which modalities can a patient self-present (without referral from a medical practitioner)?**

	N/A	Plain radiography	Radiographic contrast procedure	US	CT	MRI	Interventional radiology	PET-CT	Nuclear medicine
Patient	☐	☐	☐	☐	☐	☐	☐	☐	☐

***13. In your Member State, are there nationally recognised imaging referral guidelines (appropriateness or referral criteria) including radiation doses available?**

- ◯ Yes
- ◯ No

National Guidelines

14. The following section relates to the development of Guidelines which may not be relevant to representatives of competent authorities (regulatory bodies) who need not answer this section.

☐ Representatives of competent authorities who do not wish to answer the section on Guideline development, please tick the box to go on to the next section.

EC Survey on Imaging Referral Guidelines

National Guidelines

Respondents from competent authorities need not answer questions on this page.

15. Are these Guidelines:

- ◯ Imaging Guidelines including radiation doses issued by a single source
- ◯ Imaging Guidelines including radiation doses from multiple sources
- ◯ Clinical Guidelines with imaging guidance including radiation doses
- ◯ None of the above (please specify nature of Guidelines):

16. Please give the name of your Guidelines in English:

Main Guidelines (to which further questions refer)

Additional Guidelines (if applicable)

17. What is the source of the National Guidelines? (please select best option)

- ◯ Nationally developed
- ◯ Modified from another recognised source (please state source below)
- ◯ Adopted without modification from another recognised source (please state source below)
- ◯ Other (please state source below)

Please state source:

National Guidelines

Respondents from competent authorities need not answer questions on this page.

18. Please give the web address of your Guidelines (if available):

19. Which organisation(s) were involved in the development of your Guidelines? Please give the name(s) in English.

20. Which specialty groups took part? Please give the name(s) in English.

21. Please give the year of the first edition of your Guidelines:

22. Please indicate the approximate duration of the review cycle in years (period between editions).

National Guidelines: Financing

Respondents from competent authorities need not answer questions on this page.

23. What is the model (source) of funding (financing)? (Please indicate all that apply)

- [ ] Licensed distribution / sales
- [ ] Development grant
- [ ] Funded by Ministry/Department of Health
- [ ] Funded by other governmental department (please specify below)
- [ ] Commercial support
- [ ] Philanthropic donation
- [ ] Issuing organisation funded
- [ ] Other (please specify below)

Please specify:

National Guidelines: Scope

Respondents from competent authorities need not answer questions on this page.

24. Which imaging modalities are included in your Guidelines? (Please indicate all that apply)

- [ ] Plain radiography
- [ ] Radiographic contrast procedure
- [ ] Ultrasound
- [ ] CT
- [ ] MRI
- [ ] Interventional radiology
- [ ] Nuclear medicine
- [ ] PET-CT
- [ ] Other (please specify)

25. Is there separate guidance for children?

- ○ Yes
- ○ No
- ○ Don't know

26. Is there guidance for the pregnant woman and the unborn child?

- ○ Yes
- ○ No
- ○ Don't know

27. Which groups of diseases / medical conditions are covered for adults? (Please indicate all that apply)

- [ ] Breast
- [ ] Cancer
- [ ] Cardiovascular
- [ ] Chest
- [ ] ENT/Head and Neck
- [ ] Endocrine
- [ ] Gastrointestinal
- [ ] Gynaecology
- [ ] Musculoskeletal
- [ ] Neurological
- [ ] Obstetrics
- [ ] Trauma
- [ ] Urogenital

28. Which groups of diseases / medical conditions are covered for children? (Please indicate all that apply)

- [ ] Breast
- [ ] Cancer
- [ ] Cardiovascular
- [ ] Chest
- [ ] ENT/Head and Neck
- [ ] Endocrine
- [ ] Gastrointestinal
- [ ] Gynaecology
- [ ] Musculoskeletal
- [ ] Neurological
- [ ] Trauma
- [ ] Urogenital

29. What do your Guidelines focus on? (Please indicate all that apply)

- [ ] Clinical presentations and their imaging investigations
- [ ] Imaging procedures and their indications

National Guidelines: Development

Respondents from competent authorities need not answer questions on this page.

30. In developing an individual Guideline, which of the following were considered?

	Routinely	Occasionally	Never
Strength of evidence	○	○	○
Grading of recommendation	○	○	○
Radiation doses	○	○	○
Cost-effectiveness	○	○	○
Availability of equipment/expertise	○	○	○

31. Do you use recognised evidence levels (hierarchy of evidence) in your process?

- ○ Yes
- ○ No
- ○ Don't know

If yes please specify (eg Centre for Evidence-based Medicine [ref http://www.cebm.net/index.aspx?o=1025]; Fryback and Thornbury [ref http://www.ajronline.org/content/176/4/873.full.pdf+html]):

32. Do you apply a grading of recommendation using a recognised system?

- ○ Yes
- ○ No
- ○ Don't know

If yes please specify (eg GRADE; AHRQ [Grading ref. http://www.gradeworkinggroup.org/publications/index.htm http://www.bmj.com/content/328/7454/1490.full])

33. Was a recognised process of consensus used? (please indicate all that apply) [Consensus ref. http://www.bmj.com/content/311/7001/376?sso=]

- ☐ Delphi process
- ☐ Expert meeting for consensus
- ☐ Other (please specify):

34. Were recognised sources used for (please indicate all that apply):

- [ ] Radiation dose
- [ ] Cost-effectiveness

If yes to one or both, please specify:

35. Total number of clinical conditions/diagnostic problems (approximately):

36. If recommendations are graded, please indicate the number of:

Grade A recommendations

Grade B recommendations

Grade C recommendations

Grade D recommendations (if any)

National Guidelines: Dissemination

Respondents from competent authorities need not answer questions on this page.

37. In which format(s) are your Guidelines available?

	Free to all	Free to society members	For purchase	Not available
Print (paper copy)	☐	☐	☐	☐
Downloadable digital version	☐	☐	☐	☐
Web version (password protected)	☐	☐	☐	☐
Web version (not password protected)	☐	☐	☐	☐
PDA/tablet (app)	☐	☐	☐	☐
Smartphone (app)	☐	☐	☐	☐

38. To which of the following groups are your Guidelines routinely circulated (please indicate all that apply):

- ☐ Providers of the service (eg, radiologists, radiographers)
- ☐ Referrers (general practitioners)
- ☐ Referrers (emergency dept. clinicians)
- ☐ Referrers (specialists / hospital doctors)
- ☐ Referrers (non-medical healthcare professionals)
- ☐ Medical students
- ☐ Funders (eg, healthcare commissioners, medical insurers)
- ☐ The public

National Guidelines: Dissemination

Respondents from competent authorities need not answer questions on this page.

39. Do you advocate or recommend reinforcement of Guidelines by (please indicate all that apply):

- [ ] Periodic reminders
- [ ] Educational messages
- [ ] Other

If yes to "other" please specify:

40. Have your Guidelines been incorporated into clinical decision support systems (CDSS)?
[CDSS ref. http://www.ncbi.nlm.nih.gov/pubmed/17412171
http://www.jacr.org/article/S1546-1440(10)00389-3/abstract]

- ( ) Yes (please specify below)
- ( ) No
- ( ) Don't know

If yes, please specify:

41. For what other purposes have your Guidelines been used? (please indicate all that apply)

- [ ] For education
- [ ] By healthcare providers for planning local services
- [ ] By health insurers
- [ ] By departments of health for planning national services
- [ ] For academic/research purposes

Future Developments

In addition to the above questions member states are invited to explore how, in the future, they may wish to meet the requirements of MED 97/43 (Article 6.2: Member states shall ensure that recommendations concerning referral criteria for medical exposures, including radiation doses, are available to the prescriber of medical exposures).

Type of Guidelines

***42. Please rate the level of support of your Organisation for the following models for imaging referral guidelines.**

Rate the level of support on a scale of 1–7, where 1 is strongly oppose, 4 is neither oppose nor support and 7 is strongly support.

	1 (oppose)	2	3	4 (neutral)	5	6	7 (support)	N/A
a. Pan-European referral Guidelines developed centrally	◯	◯	◯	◯	◯	◯	◯	◯
b. European Guidelines developed by combination of multiple national Guidelines agreed by consensus	◯	◯	◯	◯	◯	◯	◯	◯
c. Adopting, modifying and translating national Guidelines from other member states	◯	◯	◯	◯	◯	◯	◯	◯
d. Independently produced national Guidelines	◯	◯	◯	◯	◯	◯	◯	◯
e. Adopting, modifying, and translating non-European Guidelines	◯	◯	◯	◯	◯	◯	◯	◯
f. Global Guidelines	◯	◯	◯	◯	◯	◯	◯	◯

Guideline format

***43. Please rate the level of support of your Organisation for the various Guideline formats listed below.**

Rate the level of support on a scale of 1–7, where 1 is strongly oppose, 4 is neither oppose nor support and 7 is strongly support.

	1 (oppose)	2	3	4 (neutral)	5	6	7 (support)	N/A
a. Narrative (plain text)	◯	◯	◯	◯	◯	◯	◯	◯
b. Tabular (tables showing initial investigations with alternatives)	◯	◯	◯	◯	◯	◯	◯	◯
c. Flowchart (box and arrow diagrams showing initial and subsequent investigations)	◯	◯	◯	◯	◯	◯	◯	◯

Media/mode for distribution

***44. Please rate the level of support of your Organisation for the media / modes for distribution listed below.**

Rate the level of support on a scale of 1–7, where 1 is strongly oppose, 4 is neither oppose nor support and 7 is strongly support.

	1 (oppose)	2	3	4 (neutral)	5	6	7 (support)	N/A
a. Print (paper copy)	◯	◯	◯	◯	◯	◯	◯	◯
b. Web version (password protected)	◯	◯	◯	◯	◯	◯	◯	◯
c. Web version (not password protected)	◯	◯	◯	◯	◯	◯	◯	◯
d. Smartphone/tablet (app)	◯	◯	◯	◯	◯	◯	◯	◯
e. Clinical decision support system (at the point of request incorporating automated, non-mandatory change of modality according to rules based on Guidelines)	◯	◯	◯	◯	◯	◯	◯	◯
f. Provision of Guidelines through electronic requesting systems (computerised order entry) as a future development	◯	◯	◯	◯	◯	◯	◯	◯

Potential barriers/challenges to the effective distribution of Guidelines

***45. Does your Organisation see the following as potential barriers/challenges to Guideline availability or use in your Member State? Please rate the level of agreement / disagreement.**

Rate the level of agreement on a scale of 1-7 where 1= strongly disagree, 4= neither agree nor disagree, and 7= strongly agree.

	1 (disagree)	2	3	4 (neutral)	5	6	7 (agree)	N/A
a. Limitation of resource (human)	◯	◯	◯	◯	◯	◯	◯	◯
b. Limitation of resource (financial)	◯	◯	◯	◯	◯	◯	◯	◯
c. Translation/language barriers	◯	◯	◯	◯	◯	◯	◯	◯
d. Dissemination / distribution barriers	◯	◯	◯	◯	◯	◯	◯	◯
e. Awareness, access and acceptability	◯	◯	◯	◯	◯	◯	◯	◯
f. Limited involvement of referring clinicians in the development process	◯	◯	◯	◯	◯	◯	◯	◯
g. Conflicting Guidelines from multiple sources	◯	◯	◯	◯	◯	◯	◯	◯
h. Lack of support or endorsement by ministries of health	◯	◯	◯	◯	◯	◯	◯	◯

Suggestions of solutions to barriers limiting the availability of Guideline...

46. Would your Organisation support the following solutions to barriers limiting the availability of Guidelines?

Rate your level of support on a scale of 1–7, where 1 is strongly oppose, 4 is neither oppose nor support and 7 is strongly support.

	1 (oppose)	2	3	4 (neutral)	5	6	7 (support)	N/A
a. Clinical decision support systems (for automated, non-mandatory change of clinican-requested modality according to rules based on Guidelines)	○	○	○	○	○	○	○	○
b. Provision of Guidelines through electronic requesting systems (computerised order entry) as a future development	○	○	○	○	○	○	○	○
c. Education (undergraduate, specialist and continuing professional education)	○	○	○	○	○	○	○	○
d. Involvement of referring clinicians	○	○	○	○	○	○	○	○
e. Other (please specify below)	○	○	○	○	○	○	○	○

Other

Preferred methods for monitoring Guideline use

47. Does your Organisation support the following methods for monitoring Guideline use?

Rate the level of support on a scale of 1–7, where 1 is strongly oppose, 4 is neither oppose nor support and 7 is strongly support.

	1 (oppose)	2	3	4 (neutral)	5	6	7 (support)	N/A
a. Local internal clinical audit	◯	◯	◯	◯	◯	◯	◯	◯
b. External clinical audit	◯	◯	◯	◯	◯	◯	◯	◯
c. Voluntary reporting	◯	◯	◯	◯	◯	◯	◯	◯
d. Inspection by regulatory body	◯	◯	◯	◯	◯	◯	◯	◯

48. Please feel free to include further comments, information or feedback (in English). We are particularly interested to hear of good practices in your Member State.

EC SURVEY ON IMAGING REFERRAL GUIDELINES IN EUROPE

Frequently Asked Questions

1. ***What is Euratom 97/43?***

   The Council Directive, Euratom 97/43 was issued in 1997 to direct Member States of the European Union in radiation protection matters. One important element of this directive is the requirement that member states shall ensure that recommendations concerning referral criteria for medical exposure, including radiation doses, are available to the prescribers of medical exposure.
   [Euratom ref. http://ec.europa.eu/energy/nuclear/radioprotection/doc/legislation/9743_en.pdf]

2. ***Who are the ESR/RCR/SFR/CIRSE/ESPR?***

   The following organisations are the partners of a consortium led by the European Society of Radiology for this European Commission (EC) project to assess availability of guidelines in Europe and to recommend future directions:

   European Society of Radiology (ESR) –
   http://www.myesr.org/cms/website.php?id=/en/eu_affairs/newfilename.htm

   Royal College of Radiologists (RCR) – http://www.rcr.ac.uk/

   La Société Française de Radiologie (SFR) - http://www.sfrnet.org/

   Cardiovascular & Interventional Radiology Society of Europe (CIRSE) – http://www.cirse.org/

   European Society of Paediatric Radiology (ESPR) - http://www.espr.org/

3. ***Where can I see examples of imaging referral guidelines?***

   Online and print copies of imaging referral guidelines can be obtained at the following web addresses

   RCR - iRefer Making the best use of clinical radiology 7e
   http://www.rcr.ac.uk/content.aspx?PageID=995

   SFR – Le Guide du bon usage des examens d'imagerie médicale
   http://www.sfrnet.org/sfr/professionnels/5-referentiels-bonnes-pratiques/guides/guide-bon-usage-examens-imagerie-medicale/guide-en-ligne/index.phtml

   EC – Radiation Protection 118
   http://ec.europa.eu/energy/nuclear/radioprotection/publication/doc/118_en.pdf

4. ***Who should complete the questionnaire?***

   The questionnaire should be completed on behalf of the organisation and should reflect the organisation's opinions, not the individual's.
   The survey does not have to be completed in one sitting; it can be saved and re-accessed via the link provided in the invitation email at any point of time until the closing date.

Please complete the survey at the SAME WORKPLACE (same IP address) and within the SAME BROWSER on which it was started to ensure correct identity.

5. ***What is the PDF version of the survey for?***

The PDF version of the survey is not for completion but is available to share with other members of your organisation for the purposes of correct completion of the web questionnaire. If you have a query or require clarification, it is useful to use the numbering of the questions in the PDF version for reference.

6. ***What are competent authorities?***

Competent authorities are the official organisations (usually governmental advisory or regulatory bodies) empowered to execute functions or advise on radiation protection issues.

7. ***Do representations of the competent authorities need to answer all questions?***

The results of the survey are highly dependent on a representative response from all European Member States (and also those countries adopting European legislation). It is essential to complete sections on guideline availability and on future developments but competent authority representatives need not answer questions on national guideline methodology and distribution.

8. ***What is self-presentation?***

Self-presentation is the presentation of an individual for an imaging procedure without a medical referral. This is often done in the context of an individual health assessment or 'whole body health screening'.

9. ***What is clinical decision support?***

Clinical decision support systems use interactive software for healthcare professionals to make choices for improved patient care. These systems usually utilise evidence-based guidance e.g. imaging referral guidelines to help referrers request appropriate imaging procedures usually through a pathway involving input of presenting clinical features in a system with limited fields.
[CDSS ref. http://www.ncbi.nlm.nih.gov/pubmed/17412171
http://www.jacr.org/article/S15461440(10)003893/abstract]

10. ***What is meant by grade of recommendation?***

The grade of recommendation is a measure of either the importance of the recommendation or the strength of evidence informing the recommendation. There are several different systems to grade recommendation.
Eg. GRADE; AHRQ [http://www.gradeworkinggroup.org/publications/index.htm
http://www.bmj.com/content/328/7454/1490.full]

Denis Remedios (denis.remedios@imperial.ac.uk)
Monika Hierath (monika.hierath@myesr.org)

ESR, March 2012
